# The diversity of educational research in the health professions: More opportunities than challenges

**DOI:** 10.3205/zma001807

**Published:** 2026-01-15

**Authors:** Katrin Schüttpelz-Brauns, Jan Matthes, Stefan Schauber, Michaela Wagner-Menghin

**Affiliations:** 1 Medical Faculty Mannheim at Heidelberg University, Division for Study and Teaching Development, Medical Education Research Department, Mannheim, Germany; 2 University of Cologne, Faculty of Medicine, Student Dean’s Office, Cologne, Germany; 3 University of Cologne, Faculty of Medicine and University Hospital, Center for Pharmacology, Cologne, Germany; 4 University of Oslo, Faculty of Medicine, Section for Health Sciences Education (HELP), Oslo, Norway; 5 Medical University of Vienna, Department of Psychiatry and Psychotherapy, Clinical Division of Social Psychiatry, Vienna, Austria; 6 Medical University of Vienna, Comprehensive Center for Clinical Neurosciences and Mental Health, Vienna, Austria

## Editorial

Researchers from disciplines such as medicine, psychology, sociology, education and the natural sciences, to name but a few, are involved in educational research in the health professions. The wide diversity of disciplines involved, with their different foundations and research methods, can be very challenging in scientific discourse. The fact that different research cultures clash here is illustrated not least by discussions on questions about what kind of knowledge should be gained [1], [2], [3], [4], [5], [6], [7], which topic and outcomes should be investigated [8], [9], [10], [11], whether and what role theories play in educational research [5], [12], [13], [14], [15], [16], which research methods should be used [5], [12], [13], [17], [18], [19], [20], [21], [22] and how study designs are assessed [8], [12], [18], [23], [24].

Since 2006, the Committee for Educational Research Methodology of the DACH Society for Medical Education (GMA) has provided a forum for the reflection and integration of different research cultures [25]. This has resulted in regular training courses on methods in educational research [26], since 2018 in the form of a winter school. With regard to the planning and implementation of educational research studies, first recommendations on ethical aspects were published in 2009 [27], to which an article in this issue refers [28]. With regard to the quality and reproducibility of the publication of research results, recommendations on writing and reviewing manuscripts for the GMS J Med Educ were published [29], [30]. In the course of the committee's work, the desire evolved over time to contribute to the discussion and mutual understanding of research cultures by editing a special issue on educational research in the health professions. The articles in the special issue are assigned to one of three focal points: Fundamentals, research methods and perspectives on educational research. 

What are the aims of educational research? This question is addressed in the article by Schüttpelz-Brauns et al. [31] in the section on the fundamentals of educational research. Using an analysis of 169 publications in pertinent journals, the authors develop a system of categories that can be used to define the topics and objectives of medical education research more precisely and to differentiate between different types of research and their relevance. Taking the theory of cognitive load as an example, Paridon [32] deals with the role of empirically tested theories in medical education research. She discusses the development of theories on the basis of findings and their contribution to the development of teaching/learning methods and materials for educational practice. The commentary by Ellaway [33] also invites reflection on one's own goals and theories against the background of the wish to further develop educational practice and educational research. She advocates reflecting on the role of philosophy in medical education. In doing so, she poses some key philosophical questions relating to medical education, the answers to which can ensure the quality and usefulness of educational research in the health professions. 

The part focusing on research methods reflects the diversity of educational research in the healthcare professions, which ultimately prompted the publication of this special issue: different types of research questions require different research methods. Articles from the "How to" category give an impression of the resulting range of methods. In their article, Giesler & Fabry [34] convey a basic understanding of the quantitative investigation of psychological characteristics such as personality traits or attitudes. They describe the development of test and questionnaire procedures, from the definition of the construct to be measured to test statistics. With the Q-method, Schick & Jedlicska [35] present a scientific procedure at the interface of qualitative and quantitative research. They do this using the example of physicians' role expectations with respect to dying and death. Ortloff et al. [36] introduce document analysis using a case study. This is usually used as part of a multi-method approach and draws on existing data and information. The article by Homberg [37] is dedicated to the Delphi method as a gold standard of consensus procedures. Her article can serve as a guideline for planning Delphi surveys and shows their possibilities and limitations. Finally, Gadewoltz [38] deals in her article with the scientific theoretical foundations and research methodological background of the Research Program Subjective Theories, whose dialogical approach opens up possibilities for systematic investigation of individual thought processes. She explains that this qualitative method not least can help to meet the challenges of interprofessional education in healthcare professions. 

In the third focus of this special issue, authors take a critical look at educational research in the healthcare professions and provide impulses for how it can be further developed. Steinberg [39] discusses the challenges of applying psychological theories and methods on learning in educational research in the healthcare professions from the perspective of educational psychology. Wijnen-Meijer & Norcini [40] comment on the sources, applications and challenges of patient-related outcome variables. Al-Buhaly’s [41] article deals with co-creation in health professions education and discusses the benefits of involving different stakeholders in the joint development of educational programs. Hirsch et al. [28] elaborate on ethical aspects of planning, conducting and publishing scientific studies, which also and possibly especially represent a challenge in educational research in the health professions. The article offers practical support in the consideration of these aspects and their implementation. 

This special issue thus not only reflects the diversity of disciplines involved in research, but also shows that this diversity and the know-how from the various research cultures can be used to answer a wide range of research questions on training and education in the healthcare professions using scientific methods. Although the research traditions face us with major challenges, we should consider this diversity as a potential and thus as an opportunity. This special issue is therefore also a call for action: let’s develop a common understanding of the different research cultures and their view of learning and teaching. By this, we can find the adequate methodology for each research question and exploit the full potential of educational research methodology in the healthcare professions.

## Authors’ ORCIDs

- Katrin Schüttpelz-Brauns: [0000-0001-9004-0724]
- Jan Matthes: [0000-0003-2754-1555]
- Stefan Schauber: [0000-0002-1832-2732]
- Michaela Wagner-Menghin: [0000-0003-1645-7577]

## Competing interests

The authors declare that they have no competing interests. 
